# Targeted Treatment Reverses Increased Left Cardiac Work in Unilateral vs. Bilateral Primary Aldosteronism

**DOI:** 10.1093/ajh/hpae087

**Published:** 2024-07-10

**Authors:** Eeva Kokko, Marianna Viukari, Jenni K Koskela, Manoj Kumar Choudhary, Niina Matikainen, Jukka Mustonen, Pasi I Nevalainen, Ilkka Pörsti

**Affiliations:** Faculty of Medicine and Health Technology, Tampere University, Tampere, Finland; Endocrinology, Helsinki University Hospital and Research Programs Unit, Clinical and Molecular Medicine, University of Helsinki, Helsinki, Finland; Faculty of Medicine and Health Technology, Tampere University, Tampere, Finland; Department of Internal Medicine, Tampere University Hospital, Tampere, Finland; Faculty of Medicine and Health Technology, Tampere University, Tampere, Finland; Endocrinology, Helsinki University Hospital and Research Programs Unit, Clinical and Molecular Medicine, University of Helsinki, Helsinki, Finland; Faculty of Medicine and Health Technology, Tampere University, Tampere, Finland; Department of Internal Medicine, Tampere University Hospital, Tampere, Finland; Department of Internal Medicine, Tampere University Hospital, Tampere, Finland; Faculty of Medicine and Health Technology, Tampere University, Tampere, Finland; Department of Internal Medicine, Tampere University Hospital, Tampere, Finland

**Keywords:** blood pressure, cardiac work, hypertension, primary aldosteronism

## Abstract

**BACKGROUND:**

The incidence of cardiovascular complications may be higher in unilateral than bilateral primary aldosteronism (PA). We compared noninvasive hemodynamics after targeted therapy of bilateral vs. unilateral PA.

**METHODS:**

Adrenal vein sampling was performed, and hemodynamics recorded using radial artery pulse wave analysis and whole-body impedance cardiography (*n* = 114). In 40 patients (adrenalectomy *n* = 20, spironolactone-based treatment *n* = 20), hemodynamic recordings were performed after 33 months of PA treatment.

**RESULTS:**

In initial cross-sectional analysis, 51 patients had bilateral and 63 unilateral PA. The mean ages were 50.6 and 54.3 years (*P* = 0.081), and body mass indexes 30.3 and 30.6 kg/m^2^ (*P* = 0.724), respectively. Aortic blood pressure (BP) and cardiac output did not differ between the groups, but left cardiac work was ~10% higher in unilateral PA (*P* = 0.022). In the follow-up study, initial and final BPs in the aorta were not significantly different, while initial cardiac output (+13%, *P* = 0.015) and left cardiac work (+17%, *P* = 0.009) were higher in unilateral than bilateral PA. After median treatment of 33 months, the differences in cardiac load were abolished, and extracellular water volume was reduced by 1.3 and 1.4 l in bilateral vs. unilateral PA, respectively (*P* = 0.814).

**CONCLUSIONS:**

These results suggest that unilateral PA burdens the heart more than bilateral PA, providing a possible explanation for the higher incidence of cardiac complications in unilateral disease. A similar reduction in aldosterone-induced volume excess was obtained with targeted surgical and medical treatment of PA.

## Graphical Abstract

**Graphical Abstract:**
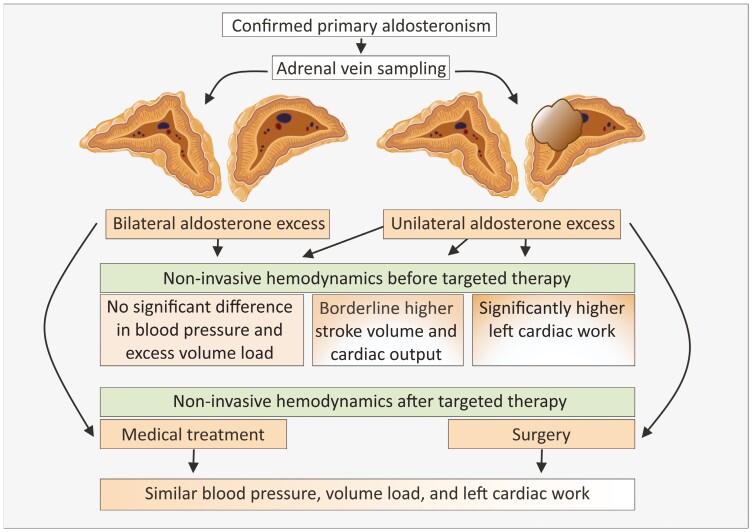

Primary aldosteronism (PA) is characterized by hypersecretion of aldosterone that is renin-independent and only partially suppressible by sodium loading.^1^ Traditionally, PA has been divided into 2 subtypes according to the origin of aldosterone excess. Unilateral PA is mainly caused by aldosterone-producing adenoma and less frequently by unilateral adrenal hyperplasia or adrenal carcinoma, whereas bilateral PA most often originates from bilateral adrenal hyperplasia.^1^ Unilateral PA has been reported to explain 30%–40% and bilateral PA 60%–70% of all PA cases.^2–4^ However, some lateralization may be detected in bilateral PA, while some degree of contralateral aldosterone secretion may be present in unilateral PA.^5^

Excessive aldosterone secretion has several unfavorable influences on the cardiovascular system. High aldosterone levels predispose individuals to sodium retention, hypokalemia, increased extracellular water (ECW) volume, and hypertension.^6,7^ In addition, aldosterone excess promotes oxidative stress and inflammation, and causes vascular, renal, and cardiac damage.^8,9^ When compared with essential hypertension, PA carries an increased risk for cardiovascular and cerebrovascular complications.^10^ Within the 2 main subtypes, unilateral PA may have a more severe hormonal profile with higher serum aldosterone and a higher aldosterone-to-renin ratio, and lower potassium levels than bilateral PA.^10^ Subsequently, patients with unilateral PA may display a higher incidence of coronary artery disease, stroke, and arrhythmias compared with those with bilateral PA.^10^

Surgical treatment of unilateral PA lowers blood pressure (BP) and reduces the risk for adverse cardiovascular outcomes.^11–13^ Medical treatment of bilateral PA using mineralocorticoid receptor antagonist (MRA) also reduces BP and the need for other antihypertensive drugs,^14–16^ but a higher risk for cardiometabolic events and even death prevails independent of BP control when compared with essential hypertension.^17^

The objective of this study was to noninvasively examine cardiovascular function in patients with confirmed unilateral vs. bilateral PA and to evaluate how the observed hemodynamic changes are corrected by targeted treatment of aldosterone excess.

## METHODS

### Study subjects

All patients participated in an ongoing investigation on the hemodynamics of primary and secondary hypertension (Eudra-CT 2006-002065-39, ClinicalTrails.gov NCT01742702).

Following a confirmed PA diagnosis, patients from all 5 University Clinics in Finland are directed to Tampere University Hospital for adrenal vein sampling (AVS).^17^ These PA patients were asked to participate in the study. AVS details have been described earlier.^18^ Exclusion criteria included symptomatic coronary artery disease, history of stroke, cardiac insufficiency, valvular heart disease, chronic kidney disease, secondary hypertension other than PA, alcohol or substance abuse, psychiatric illnesses other than mild to moderate depression or anxiety, and any heart rhythm other than sinus rhythm.

All participants were examined by a physician. Medical history, lifestyle habits, medications, smoking status, and weekly alcohol consumption were documented. Office BP measurements and laboratory analyses for elevated BP were performed according to European guidelines.^19^

In the cross-sectional part of the study, the analyses included 114 PA patients (51 with bilateral and 63 with unilateral aldosterone excess). Altogether, 40 PA patients participated in the follow-up measurements of the study. These patients were assigned to 2 groups based on the AVS results:^18^ patients with unilateral aldosterone excess to adrenalectomy (*n* = 20) and patients with bilateral aldosterone excess to spironolactone-based treatment (*n* = 20).^18^ The 2 separate hemodynamic recordings were performed 31 [27–45] months apart in the adrenalectomized group and 35 [27–53] months apart in the bilateral aldosterone excess group (*P* = 0.602).

The diagnosis of PA was based on screening and confirmatory testing.^1^ Positive PA screening results were defined as serum aldosterone (pmol/l) to plasma renin activity (ng/ml/h) ratio >750 with a serum aldosterone concentration ≥280 pmol/l; or a serum aldosterone (pmol/l) to plasma renin concentration (mU/l) ratio >30 with a serum aldosterone concentration ≥280 pmol/l.^1^ Before AVS, confirmatory testing was performed in 104 patients in the cross-sectional and 39 patients in the follow-up study, showing urine aldosterone excretion >33 nmol/day during oral sodium loading.^1^ During AVS, an aldosterone-to-cortisol ratio exceeding 4:1 (dominant vs. nondominant side) indicated unilateral PA.^18^

The study adhered to the Helsinki Declaration and was approved by the ethics committee of Tampere University Hospital (code R06086M). All participants signed an informed consent form.

### Hemodynamic measurements

A trained nurse performed hemodynamic measurements in a quiet, temperature-controlled laboratory. Participants were instructed to abstain from caffeine-containing products, smoking, and heavy meals for ≥4 hours, and from alcohol for ≥24 hours before the recordings. The patients rested supine on a table. Electrodes for impedance cardiography were attached, an oscillometric brachial cuff for BP calibration was placed in the right upper arm, and a tonometric sensor for pulse wave analysis was placed on left radial pulsation. Supine hemodynamics were recorded for 5 minutes, and the mean values of the minutes were utilized in the analyses. The good repeatability and reproducibility of the measurement protocol have been demonstrated.^20^

### Pulse wave analysis

A tonometric sensor (Colin BP-508T, Colin Medical Instruments) continually recorded BP and pulse waveform from the radial artery. The sensor was secured using a wristband. On average every 2.5 minutes, contralateral brachial BP readings were used to calibrate the BP signal from the radial artery. Aortic BP, AIx (augmentation index, i.e. augmented pressure/pulse pressure × 100), and AIx adjusted to heart rate 75 beats per minute (AIx@75) were determined using the SphygmoCor pulse wave monitoring system (PWMx, AtCor Medical, Australia).^21^

### Whole-body impedance cardiography

A whole-body impedance cardiography device (CircMon, JR Medical, Tallinn, Estonia) was utilized to measure aortic to popliteal pulse wave velocity (PWV), heart rate, stroke volume, and cardiac output throughout the cardiac cycles. The signals from the popliteal artery and whole-body impedance were recorded, and the software analyzed the time difference between these signals. PWV was calculated based on this time difference and the distance between the electrodes. To align with Doppler ultrasound-based readings, which provide more precise PWV measurements, a validated equation was applied to adjust the values derived from impedance cardiography (PWV = (PWV impedance × 0.696) + 0.864).^22^

The BP data from the radial tonometric sensor and the cardiac index determined by CircMon were used to calculate the systemic vascular resistance index. The procedure and electrode arrangement have been previously documented.^22–24^

### Laboratory tests

Blood and urine samples were obtained after ~12 hours of fasting. Electrolyte, glucose, cystatin-C, lipid, C-reactive protein, uric acid, and creatinine concentrations were measured using Cobas Integra 700/800 (F. Hoffmann-Laroche Ltd, Basel; Switzerland) or Cobas 6000, module c501 (Roche Diagnostics, Basel, Switzerland). The ADVIA 120 or 2120 (Bayer Health Care, Tarrytown, NY) was used for blood cell counting. The quantities of aldosterone were measured on API 4000 (Sciex) using liquid chromatography–mass spectrometry (LC–MS/MS), as previously described, with intra- and inter-assay coefficients of variation ≤5.8% and ≤7.5%, respectively.^25,26^

Plasma renin activity was initially assessed using radioimmunoassay (DiaSorin, Saluggia, Italy), and during recruitment, it was replaced by determination of direct renin concentration (LIAISON immunoanalyzer, DiaSorin, Saluggia, Italy). The intra- and inter-assay coefficients of variation in the laboratory were ≤5.8% and ≤11.2% for plasma renin activity, and ≤5.6% and ≤12.4% for renin concentration, respectively. The low detection limits of the analyses (0.2 ng/ml/h for plasma renin activity and 2 mU/l for renin concentration) were reported for patients with extremely low renin levels. Patients with overt kidney disorders were excluded based on urine dipstick refractometer studies (Siemens Clinitec Atlas or Advantus, Siemens Healthcare GmbH, Erlangen, Germany), as well as plasma creatinine and cystatin-C concentrations.

### Statistical analysis

The one-way analysis of variance was used to analyze demographic and laboratory data with normal distribution. For data that were not normally distributed, the Kruskal–Wallis test with *post hoc* Mann–Whitney *U* test was applied. Generalized estimating equation analyses were used to examine hemodynamic variables across the groups over the 5 minutes of repeated observations. In the cross-sectional study, the groups exhibited variations in body mass index, number of diabetics, age, and cystatin-C concentration. These variables were included as covariates in the generalized estimating equation analyses.

The results were presented as mean (SD) if normally distributed or as median [25^th^–75^th^ percentile] if non-normally distributed. Categorial variables were expressed as numbers. *P* < 0.05 was considered significant. All data were analyzed using SPSS version 28.0 (IBM SPSS Statistics, Armonk, NY).

## RESULTS

### Study population, laboratory values, and medications

Seventy-five (66%) male and 39 (34%) female subjects participated in the cross-sectional study (Table 1). Age ranged from 22 to 73 years, and body mass index, prevalence of type 2 diabetics, smoking habits, and alcohol consumption did not differ between the groups. BP levels in the office were also corresponding. Blood hemoglobin, and plasma concentrations of C-reactive protein, creatinine, high-density lipoprotein, and low-density lipoprotein cholesterol, triglycerides, and glucose values were similar between the groups. However, plasma potassium was 0.4 mmol/l lower and plasma sodium 1.3 mmol/l higher in the unilateral group.

**Table 1.** Cross-sectional study: demographic and clinical characteristics in bilateral and unilateral aldosterone excess

	Bilateral (*n* = 51)	Unilateral (*n* = 63)
Male/female (*n*)	32/19	43/20
Age (y)	50.6 (12.2)	54.3 (10.0)
Body mass index (kg/m^2^)	30.3 (4.9)	30.6 (5.3)
Type 2 diabetes (*n*)	10	17
Alcohol (standard drinks/wk)	2 [0–5]	3 [1–5]
Current smokers (*n*)	11	10
Office measurements		
Systolic blood pressure (mm Hg)	151 (19)	157 (15)
Diastolic blood pressure	91 (11)	92 (11)
Blood hemoglobin (g/l)	146 (14)	147 (11)
C-reactive protein (mg/l)	1.4 [0.5–3.1]	1.4 [0.5–3.4]
Potassium (mmol/l)	3.7 (0.3)	3.3 (0.4)^*^
Sodium (mmol/l)	142.0 (2.1)	143.3 (2.2)^*^
Creatinine (µmol/l)	78 (21)	77 (22)
Total cholesterol (mmol/l)	4.5 (1.0)	4.7 (0.9)
HDL cholesterol (mmol/l)	1.3 (0.4)	1.4 (0.5)
LDL cholesterol (mmol/l)	2.9 (0.9)	3.0 (0.9)
Triglycerides (mmol/l)	1.3 [0.8–1.7]	1.2 [0.9–1.8]
Glucose (mmol/l)	6.3 (1.1)	6.6 (1.4)

Mean (SD) or median [25^th^–75^th^ percentile]. Abbreviations: HDL, high-density lipoprotein; LDL, low-density lipoprotein.

^*^ *P* < 0.05 vs. bilateral.

The laboratory findings of PA are shown in Table 2. The lateralization index, calculated from the aldosterone-to-cortisol ratio during AVS,^18^ differentiated between the unilateral and bilateral groups. The unilateral group exhibited a more severe profile than the bilateral group: the lowest plasma potassium was 0.3 mmol/l lower, and the highest urinary potassium excretion was 37 mmol/24 hours higher in the former. Additionally, serum aldosterone, aldosterone-to-renin activity ratio, aldosterone-to-renin concentration ratio, and urinary aldosterone excretion were higher, and plasma renin activity was lower, in the unilateral than in the bilateral group. Urinary sodium excretion did not differ between the groups.

**Table 2.** Cross-sectional study: laboratory data of primary aldosteronism in bilateral and unilateral aldosterone excess

	Bilateral	Number^a^	Unilateral	Number^a^	Normal range
Adrenal vein sampling: lateralization index	1.5 [1.2–2.1]	51	12.4 [5.5–39.5]^***^	63	Not applicable
Lowest plasma potassium (mmol/l)	3.3 [3.1–3.6]	51	2.9 [2.7–3.1]^**^	63	3.3–4.8
Serum aldosterone (pmol/l)	578 [424–858]	51	779 [522–1,053]^*^	63	<520
Plasma renin activity (ng of Ang I/ml/h)	0.2 [0.2–0.3]	29	0.2 [0.2–0.2]^**^	43	1.5–5.7
Plasma renin concentration (mU/l)	10.6 [5.4–27.3]	22	5.0 [5.0–9.8]^**^	20	4.4–46
Ratio of aldosterone-to-renin activity	1,880 [1,390–2,910]	29	3,690 [1,770–4,985]^**^	43	<750
Ratio of aldosterone-to-renin concentration	52 [33–99]	22	111 [57–161]^**^	20	<30
Urinary aldosterone (nmol/24 h)	58 [45–76]	47	70 [51–107]^*^	57	<40
Urinary sodium (mmol/24 h)	232 [178–261]	41^b^	224 [171–281]	43^b^	130–240
Highest urinary potassium (mmol/24 h)	91 [77–119]	37	119 [99–148]^**^	38	60–90

Values are median [25^th^–75^th^ percentile].

^a^Number of patients with available results of the laboratory determination.

^b^All referring hospitals did not provide information about sodium excretion although aldosterone excretion was given.

^*^ *P* < 0.05 vs. bilateral.

^**^ *P* < 0.01.

^***^ *P* < 0.001.

Thirty (75%) male and 10 (25%) female participants comprised the follow-up population (Table 3). Age, body mass index, number of type 2 diabetics and current smokers, office systolic and diastolic BP, heart rate, and laboratory values were similar between the groups, except for a moderately higher alcohol intake (median 4 vs. 1 standard drinks/week) and a 0.3 mmol/l lower initial plasma potassium concentration in the unilateral compared with the bilateral group. Consistent with the cross-sectional study, the lowest plasma potassium was 0.4 mmol/l lower, and the highest urinary potassium excretion was 42 mmol/24 hours higher in the unilateral group at the beginning of the follow-up (Table 4). At the end of the study, plasma potassium did not differ between the groups (Table 3).

**Table 3.** Follow-up study: demographic and clinical characteristics in bilateral and unilateral aldosterone excess

	Bilateral (*n* = 20)	Unilateral (*n* = 20)
Male/female (*n*)	14/6	16/4
Age (y)	53.1 (10.3)	57.3 (7.5)
Height (cm)	177.5 (10.9)	176.7 (6.2)
Weight (kg)	92.0 (16.9)	100.0 (20.5)
Body mass index (kg/m^2^)	29.2 (4.8)	31.8 (5.2)
Type 2 diabetes (*n*)	5	7
Alcohol (standard drinks/wk)	1 [0–3]	4 [1–6]^*^
Current smokers (*n*)	3	3
Office measurements		
Systolic blood pressure (mm Hg)	155 (16)	153 (16)
Diastolic blood pressure	93 (12)	92 (13)
Heart rate (bpm)	67 (14)	68 (11)
Blood hemoglobin (g/l)	148 (13)	150 (12)
C-reactive protein (mg/l)	1.1 [0.5–1.7]	1.3 [0.5–2.5]
Potassium (mmol/l)		
Initial	3.7 (0.4)	3.4 (0.5)^*^
Final	4.0 (0.4)	4.2 (0.3)
Sodium (mmol/l)	142.4 (2.3)	142.9 (2.6)
Cystatin-C (mg/l)	0.97 (0.17)	1.03 (0.19)
Creatinine (µmol/l)	81 (17)	77 (11)
Total cholesterol (mmol/l)	4.5 (1.2)	4.7 (1.0)
HDL cholesterol (mmol/l)	1.4 (0.5)	1.3 (0.5)
LDL cholesterol (mmol/l)	2.9 (1.0)	3.1 (0.9)
Triglycerides (mmol/l)	1.4 [0.7–1.7]	1.3 [1.0–1.9]
Glucose (mmol/l)	6.1 (0.7)	6.7 (1.4)

Mean (SD) or median [25^th^–75^th^ percentile]. Abbreviations: HDL, high-density lipoprotein; LDL, low-density lipoprotein.

^*^ *P* < 0.05 vs. bilateral.

**Table 4.** Follow-up study: laboratory data of primary aldosteronism in bilateral and unilateral aldosterone excess

	Bilateral	Number^a^	Unilateral	Number^a^	Normal range
Adrenal vein sampling: lateralization index	1.6 [1.3–2.3]	51	30.3 [6.9–76.6]^***^	63	Not applicable
Lowest plasma potassium (mmol/l)	3.3 [3.0–3.6]	20	2.9 [2.7–3.1]^***^	20	3.3–4.8
Serum aldosterone (pmol/l)	509 [429–928]	20	707 [422–914]	20	<520
Plasma renin activity (ng of Ang I/ml/h)	0.2 [0.2–0.3]	12	0.2 [0.2–0.2]	17	1.5–5.7
Plasma renin concentration (mU/l)	8.6 [5.0–24.6]	8	5.1 [5.0–13.0]	3	4.4–46
Ratio of aldosterone-to-renin activity	2,490 [1,606–3,471]	12	3,795 [1,950–4,630]	17	<750
Ratio of aldosterone-to-renin concentration	65 [33–97]	8	135 [34–144]	3	<30
Urinary aldosterone (nmol/24 h)	59 [53–68]	20	70 [45–104]	19	<40
Urinary sodium (mmol/24 h)	230 [178–254]	20	245 [173–281]	20	130–240
Highest urinary potassium (mmol/24 h)	90 [80–110]	16	132 [113–154]^***^	18	60–90

Values are median [25^th^–75^th^ percentile].

^a^Number of patients with the available result of the laboratory determination.

^***^ *P* < 0.001 vs. bilateral.

At the beginning of the follow-up, the average number and defined daily doses of antihypertensive agents were similar in the bilateral and unilateral groups (Supplementary Table S1 online). After 31–35 months of follow-up, the number and doses of antihypertensive medications were significantly reduced in the unilateral group.

### Hemodynamics

**Figure 1.**
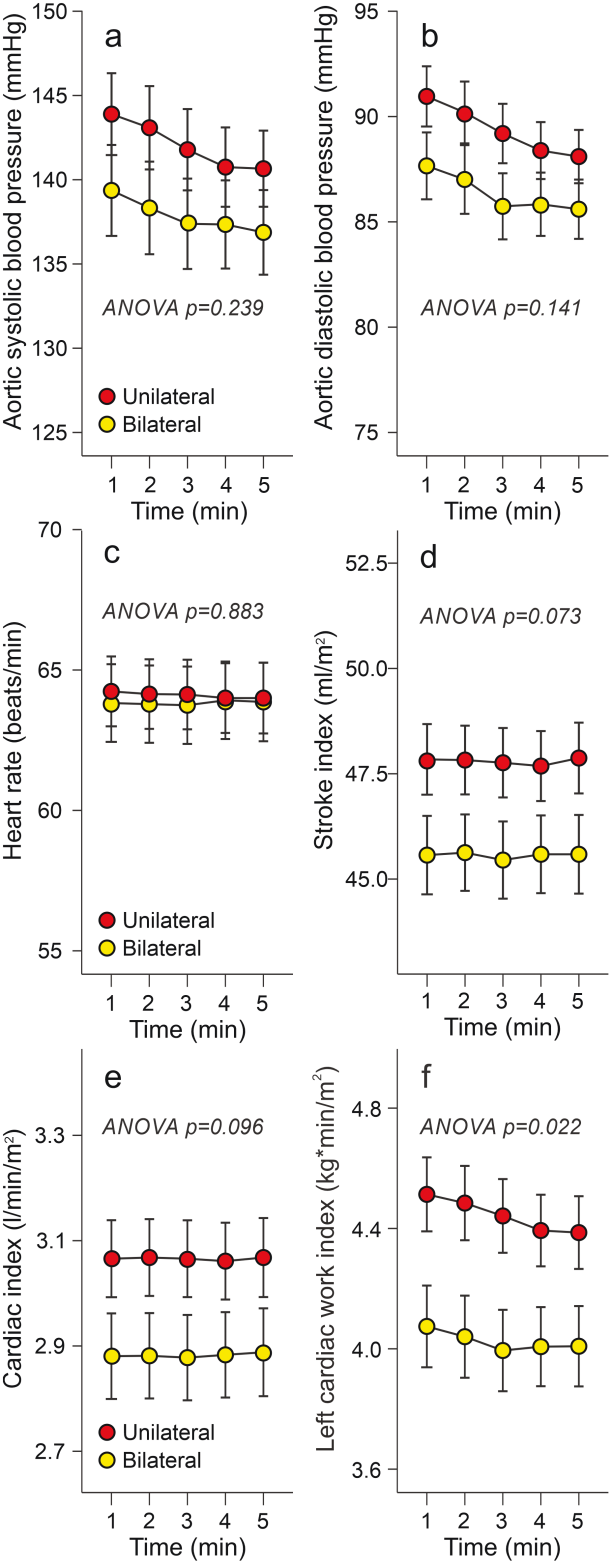
Cross-sectional study: 5-minute recordings show aortic systolic (**a**) and diastolic (**b**) blood pressure, heart rate (**c**), stroke index (**d**), cardiac index (**e**), and left cardiac work index (**f**) among 51 patients with bilateral and 63 patients with unilateral primary aldosteronism.

In the cross-sectional study, aortic systolic and diastolic BP, heart rate, stroke index, and cardiac index did not differ between the groups (Figure 1a–e). The only hemodynamic difference was a higher left cardiac work index in unilateral PA (Figure 1f).

**Figure 2.**
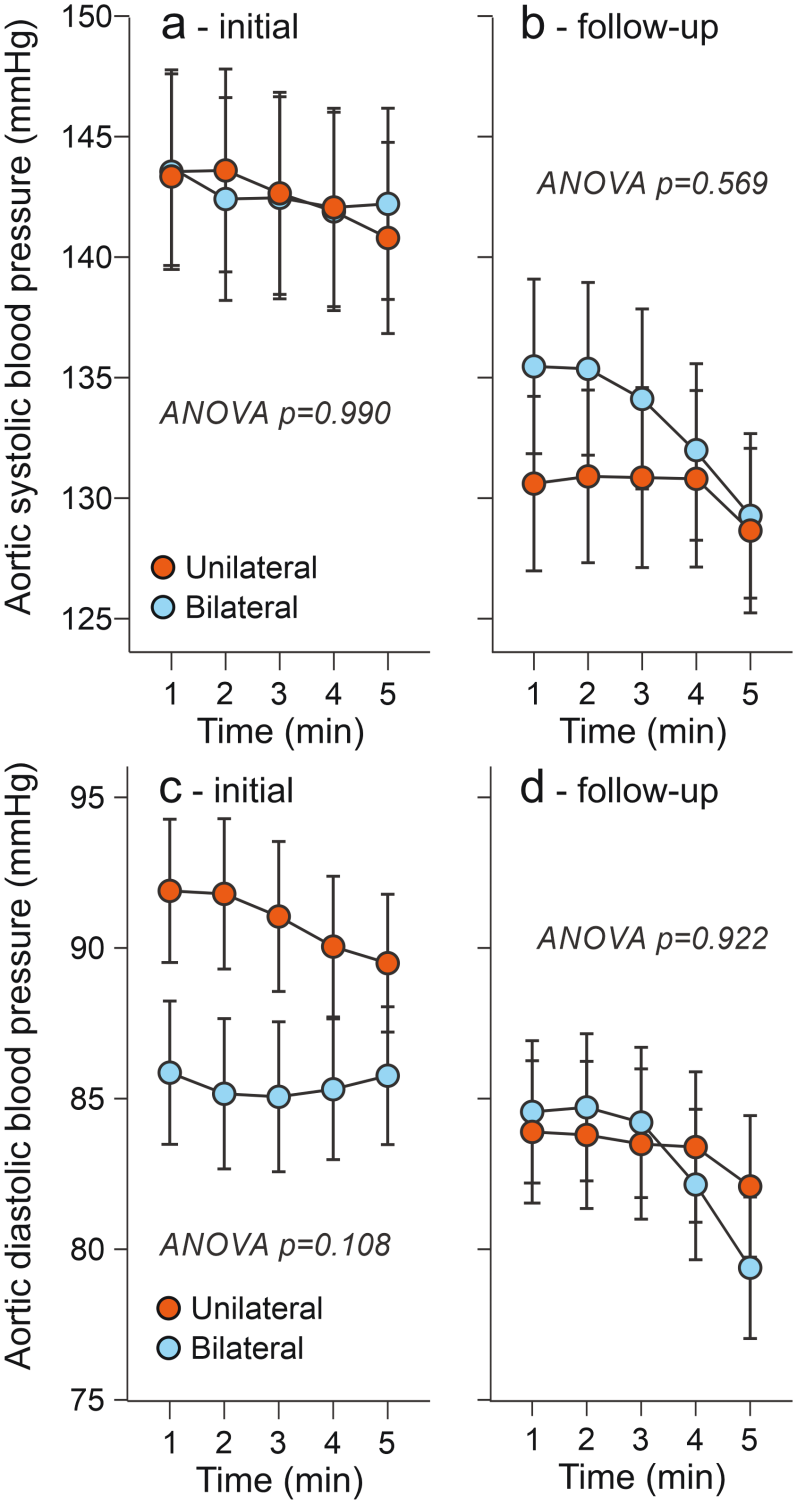
Follow-up study: 5-minute recordings show aortic systolic and diastolic blood pressure in 20 patients with bilateral and 20 patients with unilateral primary aldosteronism before (**a**, **c**) and after (**b**, **d**) 31–35 months of targeted treatment.

**Figure 3.**
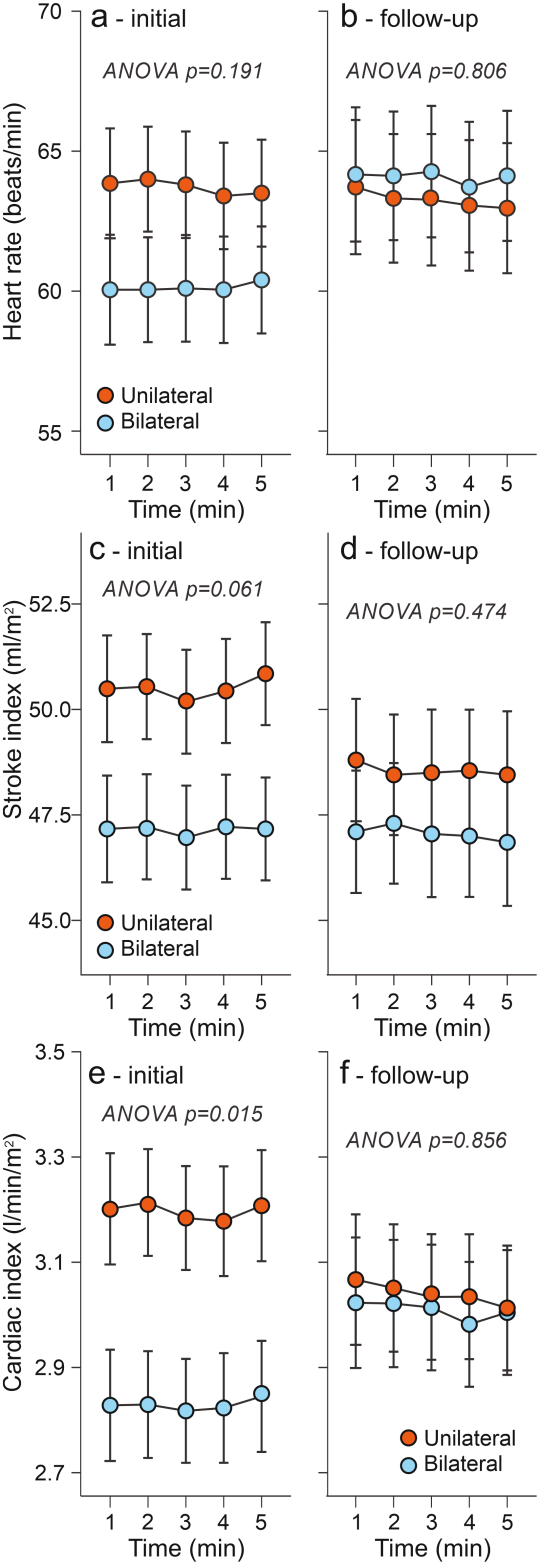
Follow-up study: heart rate, stroke index, and cardiac index in 20 patients with bilateral and 20 patients with unilateral primary aldosteronism before (**a**, **c**, **e**) and after (**b**, **d**, **f**) 31–35 months of targeted treatment.

**Figure 4.**
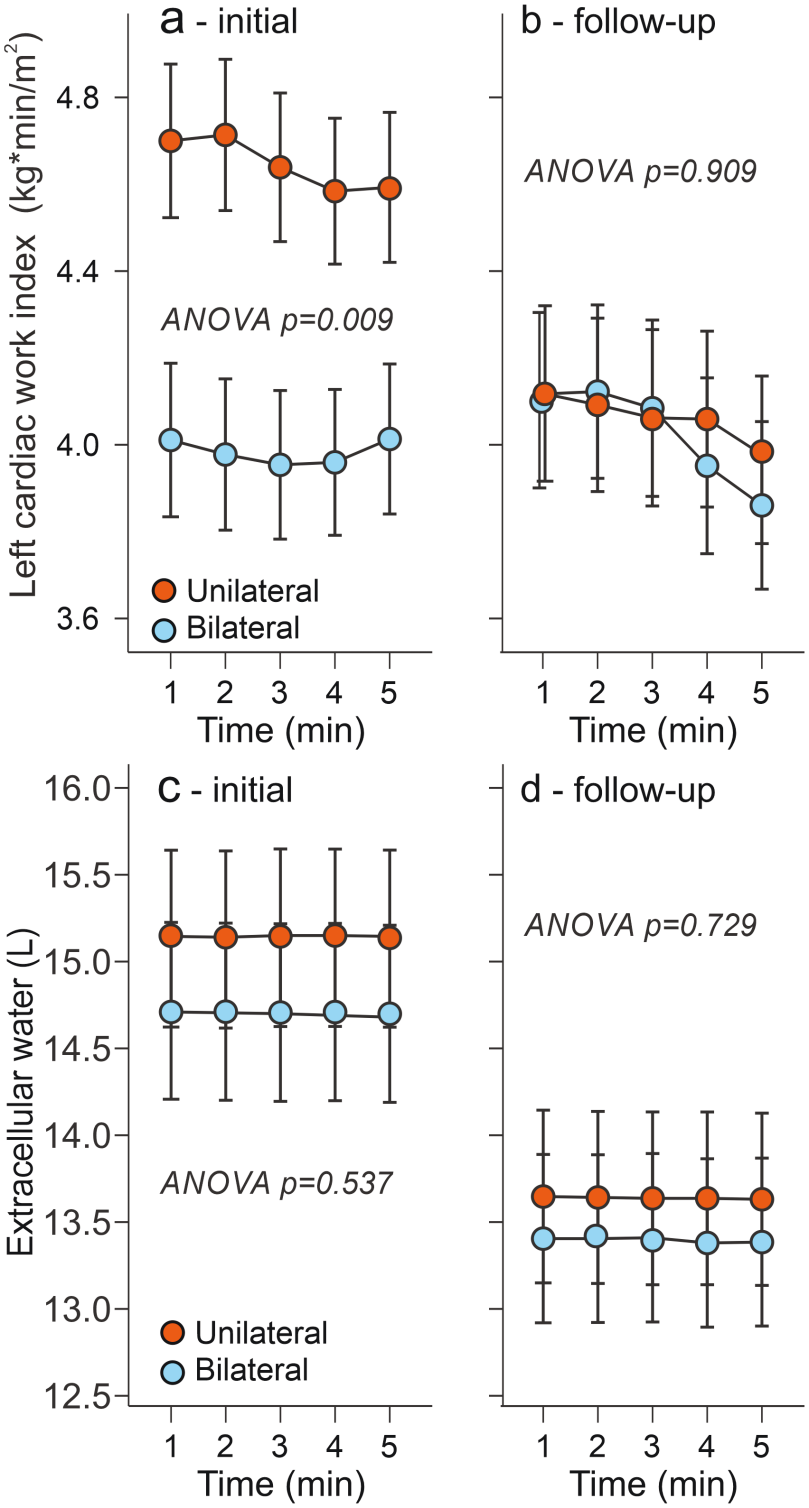
Follow-up study: left cardiac work index and extracellular water volume in 20 patients with bilateral and 20 patients with unilateral primary aldosteronism before (**a**, **c**) and after (**b**, **d**) 31–35 months of targeted treatment.

In the follow-up study, initial and final aortic systolic and diastolic BPs were similar between the groups (Figure 2a–d). Initial and final heart rate and stroke index did not differ in the groups, either (Figure 3a–d). However, cardiac index and left cardiac work index were higher in the unilateral than in the bilateral aldosteronism group at the beginning of the follow-up (Figures 3e and 4a). After targeted treatment of PA, the cardiac index and left cardiac work index in the unilateral group were clearly reduced and no longer differed from those in the bilateral group (Figures 3f and 4b). Consistent with recent findings,^27,28^ both the unilateral and bilateral groups were initially characterized by excess ECW volume (Figure 4c). After a median treatment time of 33 months, the excess ECW was equally reduced by adrenalectomy and medical treatment (by 1.4 and 1.3 l, respectively, Figure 4d).

## DISCUSSION

In this study, we compared the hemodynamic characteristics of unilateral and bilateral PA. All participants were confirmed to have PA^1^ and underwent AVS to study lateralization.^18^ Except for plasma potassium and sodium concentrations, all other laboratory values were similar in the unilateral and bilateral PA groups. In the cross-sectional study of 114 participants, small numerical, nonsignificant deviations were observed in aortic BP, stroke index, and cardiac index. However, these deviations resulted in higher left cardiac work in unilateral compared with bilateral PA, as calculated from mean aortic BP and cardiac output.^29,30^ The follow-up study with 40 participants showed similar findings: initial aortic diastolic BP and stroke index were numerically higher but not significantly different in unilateral aldosteronism, while both cardiac index and left cardiac work were higher in unilateral compared with bilateral aldosteronism. In the follow-up measurements, targeted treatment over a median follow-up time of 33 months abolished the difference in left cardiac work and reduced ECW volume by 1.3–1.4 l.

Unilateral PA may manifest at a younger age and with a more severe clinical phenotype compared with bilateral PA.^2,3^ Patients with unilateral PA also face a higher risk of adverse cardiovascular outcomes, including coronary artery disease, stroke, and arrhythmias, compared with those with bilateral PA.^10^ However, previous studies comparing hemodynamics between unilateral and bilateral PA are scarce. In the present cross-sectional study, the only differing hemodynamic variable between groups was the left cardiac work index, which was higher in the unilateral group. Initially, in the follow-up study, the unilateral group showed a higher left cardiac work index and cardiac index. These burdens of the heart were alleviated during the follow-up, resulting in similar final values in both groups. This finding may partially explain previous reports showing no difference in the incidence of cardiovascular events between unilateral and bilateral PA after aldosterone excess is reversed by adrenalectomy or MRA treatment.^31^

Initial brachial BPs in the office and aortic BPs in the laboratory, and final aortic BPs in the laboratory, were not significantly different between unilateral and bilateral aldosterone excess groups. However, the unilateral PA group exhibited a more severe biochemical profile with higher ARR and plasma sodium, and lower plasma potassium concentration, and increased 24-hour urinary potassium excretion compared with the bilateral PA group. After adrenalectomy and MRA treatment, plasma potassium levels increased, abolishing the difference in plasma potassium concentration. In the follow-up study, alcohol consumption was slightly higher in the unilateral group compared with the bilateral group. However, according to Finnish guidelines,^32^ average alcohol consumption was within an acceptable range in both groups.

Recent studies have indicated that surgical treatment, the cornerstone of unilateral PA management, is superior to medical treatment in reversing PA’s cardiovascular complications.^11,17,33,34^ However, AVS was not universally performed in these studies, thus the reliable distinction between unilateral and bilateral PA subtypes was suboptimal, affecting the generalizability of the results. According to a retrospective cohort of 2202 PA patients, adrenalectomy reduced the incidence of new-onset atrial fibrillation compared with matched hypertensive controls, while this protective effect was absent in PA patients treated with MRAs.^33^ Of note, the renin status of PA patients during MRA treatment may influence prognosis.^17,35^ After ~8 years of follow-up among 195 MRA-treated and 201 adrenalectomized patients, the risk of developing atrial fibrillation did not differ from essential hypertension in adrenalectomized PA patients and MRA-treated PA patients with non-suppressed renin.^35^ However, the hazard ratio for atrial fibrillation was 2.55-fold higher in MRA-treated PA patients with persistently suppressed renin.^35^ Compared with essential hypertension, PA patients with persistently suppressed renin during MRA treatment had an increased risk for cardiovascular events (hazard ratio 2.83) and mortality (hazard ratio 1.79), while those with unsuppressed renin did not show an elevated risk of these endpoints.^17^ This analysis comprised 602 MRA-treated PA patients.^17^ A systematic review of 16 papers found similar clinical outcomes between PA patients treated with adrenalectomy or MRA, with adrenalectomized patients showing a greater reduction in the number of antihypertensive medications over time.^34^ Following PA treatment, left ventricular mass was efficiently reduced with both approaches, with surgical treatment showing earlier effects than MRA treatment.^11^

In the present follow-up study, approximately 1.3–1.4 l reduction in ECW volume was observed in both treated PA groups. This aligns with our previous findings showing that bioimpedance measurements can be used to evaluate ECW volume in PA,^27,28^ and that targeted treatment normalizes volume excess.^28^ In patients with unilateral aldosterone excess, adrenalectomy led to earlier increases in sodium excretion and faster reductions in excess ECW volume compared with MRA treatment.^36^ Although treatment-resistant hypertension is considered the primary phenotypic indicator of PA, it is valuable to consider concurrent changes in left cardiac work and ECW volume when evaluating the clinical significance of aldosterone excess.

Our study has some limitations. The study groups were relatively small, and the results should be extrapolated cautiously. Although follow-up data on BP, volume status, plasma potassium concentration, and medications used by the participants were recorded, the consensus criteria of the Primary Aldosteronism Surgery Outcome (PASO) investigators were not fully adhered to, as serum aldosterone and plasma renin concentrations were not analyzed at the end of the study.^13^ The small differences in alcohol consumption among the follow-up groups could have influenced BP, given the direct linear correlation between alcohol use and systolic BP, without an apparent threshold in the relationship.^37^ The sex distribution within the follow-up groups, albeit not statistically significantly different, was not identical, with 70% and 80% of patients being male in the bilateral and unilateral groups, respectively. However, we have no reason to suspect that targeted treatment of PA would dissimilarly affect volume status in male and female patients. The direct renin concentration was measured in a subset of participants, whereas plasma renin activity was determined in most patients. Nonetheless, this variation should not affect the interpretation of the results, given that confirmatory testing for PA was performed in over 91% of the participants. Moreover, the division into study groups was based on the results of the AVS procedure, specifically the aldosterone-to-cortisol ratios. Finally, hemodynamics were assessed using indirect noninvasive methods, where stroke volume and cardiac output were derived from the bioimpedance signal^38^ and central aortic waveform from the applanation tonometry signal.^21^ While the approaches have been verified against direct or invasive measures, care must be applied when interpreting the results.^22,23,39^

In conclusion, the present results indicate that patients with unilateral PA exhibit a more severe hemodynamic load on the heart compared with those with bilateral PA. Surgical treatment leads to a significant reduction in cardiac workload and eliminates the gap between the PA subtypes. Our findings underscore the importance of distinguishing between the subtypes of PA to select the correct treatment modality to mitigate the deteriorating effects of aldosterone excess.

## SUPPLEMENTARY DATA

Supplementary materials are available at *American Journal of Hypertension* (http://ajh.oxfordjournals.org).

hpae087_suppl_Supplementary_Materials

## Data Availability

The data to support the findings of this study are available from the corresponding author, upon reasonable request.
