# Supplementary material for: Targeted Treatment Reverses Increased Left Cardiac Work in Unilateral vs. Bilateral Primary Aldosteronism
Source: Am J Hypertens. 2024 Jul 10;37(11):884–92. doi: 10.1093/ajh/hpae087 (PMC11471835; doi:10.1093/ajh/hpae087)
Supplement: hpae087_suppl_Supplementary_Materials [file hpae087_suppl_supplementary_materials.docx]

**Supplemental Table S1.** Number and doses of antihypertensives, and number of subjects using listed medications.

|  | **In the beginning** | | **After** **31-35 months of follow-up** | |
| --- | --- | --- | --- | --- |
|  | Bilateral aldosteronism | Unilateral aldosteronism | Bilateral aldosteronism | Unilateral aldosteronism |
| Number of antihypertensive agents | 3.1 (1.6) | 3.0 (1.3) | 3.5 (1.5) | 2.0 (1.6)** |
| Defined daily doses | 4.6 (2.8) | 5.1 (2.2) | 4.1 (2.5) | 2.7 (2.5)** |
| Potassium supplement | 10 | 14 | 1 | 0 |
| ACE inhibitor | 5 | 5 | 4 | 4 |
| Angiotensin II receptor blocker | 9 | 12 | 9 | 6 |
| Beta blocker | 12 | 9 | 10 | 8 |
| Beta and alpha blocker | 1 | 4 | 0 | 2 |
| Calcium channel blocker | 18 | 18 | 16 | 13 |
| Thiazide | 4 | 2 | 3 | 2 |
| Furosemide | 2 | 1 | 3 | 1 |
| Spironolactone | 0 | 1 | 19 | 2 |
| Moxonidine | 2 | 4 | 1 | 0 |
| Minoxidil | 0 | 1 | 0 | 0 |
| Prazosin | 19 | 5 | 4 | 1 |
| Statin | 9 | 7 | 10 | 7 |
| Acetylsalicylic acid (100 mg/day) | 5 | 6 | 5 | 7 |
| Metformin | 5 | 4 | 5 | 4 |
| Incretin mimetics | 0 | 1 | 0 | 2 |
| Dipeptidyl-peptidase 4 inhibitor | 2 | 2 | 3 | 2 |
| Sulfonylureas | 0 | 1 | 0 | 0 |
| Insulin | 1 | 3 | 0 | 0 |
| Antihistamines | 1 | 1 | 1 | 1 |
| 5α-reductase inhibitors | 0 | 1 | 0 | 0 |
| Warfarin | 2 | 0 | 2 | 0 |
| Allopurinol | 0 | 0 | 0 | 1 |
| Benzodiazepines | 0 | 1 | 1 | 1 |
| Antidepressants | 1 | 4 | 1 | 2 |
| Antiepileptics | 1 | 0 | 1 | 1 |
| Non-steroidal anti-inflammatory drugs | 1 | 1 | 0 | 0 |
| Coxibs | 0 | 1 | 0 | 0 |
| Antiarrhythmic agents | 0 | 1 | 0 | 1 |
| Vitamin D supplement | 2 | 3 | 3 | 2 |
| Calcium supplement | 1 | 2 | 2 | 2 |
| Proton pump inhibitor | 0 | 1 | 1 | 0 |
| Thyroxin | 1 | 0 | 0 | 2 |

Mean (SD); ^**^p<0.01 vs. 1^st^ visit.
